# Protection against the New Equine Influenza Virus Florida Clade I Outbreak Strain Provided by a Whole Inactivated Virus Vaccine

**DOI:** 10.3390/vaccines8040784

**Published:** 2020-12-21

**Authors:** Sylvia Reemers, Sander van Bommel, Qi Cao, David Sutton, Saskia van de Zande

**Affiliations:** 1 MSD Animal Health, Wim de Körverstraat 35, 5831 AN Boxmeer, The Netherlands; sander.vanbommel@merck.com (S.v.B.); qi.cao2@merck.com (Q.C.); saskia.vandezande@merck.com (S.v.d.Z.); 2 MSD Animal Health, Walton Manor, Walton, Milton Keynes MK7 7AJ, UK; david.sutton@msd.com

**Keywords:** equine influenza, H3N8, vaccine, immunity, protection

## Abstract

Equine influenza virus (EIV) is a major cause of respiratory disease in horses. Vaccination is an effective tool for infection control. Although various EIV vaccines are widely available, major outbreaks occurred in Europe in 2018 involving a new EIV H3N8 FC1 strain. In France, it was reported that both unvaccinated and vaccinated horses were affected despite >80% vaccination coverage and most horses being vaccinated with a vaccine expressing FC1 antigen. This study assessed whether vaccine type, next to antigenic difference between vaccine and field strain, plays a role. Horses were vaccinated with an ISCOMatrix-adjuvanted, whole inactivated virus vaccine (Equilis Prequenza) and experimentally infected with the new FC1 outbreak strain. Serology (HI), clinical signs, and virus shedding were evaluated in vaccinated compared to unvaccinated horses. Results showed a significant reduction in clinical signs and a lack of virus shedding in vaccinated horses compared to unvaccinated controls. From these results, it can be concluded that Equilis Prequenza provides a high level of protection to challenge with the new FC1 outbreak strain. This suggests that, apart from antigenic differences between vaccine and field strain, other aspects of the vaccine may also play an important role in determining field efficacy.

## 1. Introduction

A major cause of viral respiratory disease in horses is equine influenza virus (EIV). Vaccination remains the most effective method to limit EIV infection and spread and reduce outbreaks [1].

Influenza viruses are known for their high mutation rate, causing antigenic drift over time, which is also visible in the phylogenetic history of EIV [1,2]. This may result in a decrease in protection against a new field strain provided by a vaccine strain. Therefore, the OIE expert panel on EIV vaccines annually reviews antigenic differences in field strains and, based on this, advises on vaccine strain composition. EIV strains of the Florida clade (FC) 1 and 2 sub-lineages are predominant in the field nowadays, and were included in the recommendations for EIV vaccines of the OIE in 2013 [1,3].

From early 2018 to late 2018/early 2019, major EIV outbreaks were reported in South America and a great part of Europe [4,5,6]. Genetic analysis revealed the outbreak strains belonged to the FC1 sub-lineage, which is present in most commercially available vaccines. In most countries with a high vaccination rate, the number of outbreaks was low. However, in France, many outbreaks still occurred, despite a very high vaccination rate with the vast majority of horses vaccinated with a fully-updated vaccine in line with OIE recommendations [4,7]. This raises the question of whether other attributes of EIV vaccines, such as whether the antigen is whole virus or subunit, and the type of adjuvant may be equally important factors affecting the performance of vaccines in the field.

To this end, this paper presents the assessment of the efficacy of Equilis Prequenza following challenge with an isolate of the recent FC1 outbreak strain in horses.

## 2. Materials and Methods

### 2.1. Horses

Ten six-month old Shetland ponies seronegative for EIV (based on screening during acclimatization) were included in the study. Upon inclusion, horses were housed in one room at the study site one in which they remained until the day of challenge. On the day of challenge, horses were transported to a second study site and divided into two rooms by the study director, with each room containing an equal distribution of both treatment groups.

### 2.2. Vaccine

In this study, a commercially available vaccine was used: Equilis Prequenza (MSD Animal Health; R&D lot 15A19), an ISCOMatrix (purified saponin, cholesterol, and phosphatidylcholine) adjuvanted, whole inactivated virus vaccine containing EIV strains A/equine/South Africa/4/03 (FC1 sub-lineage) and A/equine/Newmarket/2/93 (European lineage). The vaccine was used within the shelf-life of the product.

### 2.3. EIV Viruses

An isolate of virus collected from an outbreak in the Netherlands on 25 December 2018 (EIV H3N8 A/equine/Venlo/19 (FC1 sub-lineage) was grown in embryonated hens’ eggs, purified and titrated as described previously [8], and used for the experimental challenge infection of the ponies. EIV H3N8 strains A/equine/South Africa/4/03 (FC1 sub-lineage), A/equine/Ohio/03 (FC1 sub-lineage), A/equine/Shropshire/10 (FC2 sub-lineage), and A/equine/Newmarket/2/93 (European lineage) were used as hemaglutination inhibition (HI) antigens.

### 2.4. Vaccination and Challenge Infection Protocol

Horses were divided randomly into two treatment groups. Group 1 (*n* = 5) received Equilis Prequenza and group 2 (*n* = 5) remained unvaccinated and served as control group. Each horse from group 1 was vaccinated intramuscularly (21 G × 1½, 0.8 × 40 mm needle) with 1 mL of the vaccine (single dose) on day 0 (V1) in the left side of the neck and on day 28 (week 4, V2) in the right side of the neck. All horses were challenge infected with EIV H3N8 A/equine/Venlo/19 (FC1 sub-lineage) on day 49 (week 7). For this purpose, 3 mL of phosphate-buffered saline-diluted allantoic fluid containing a target titre of 10^7.2^ egg infectious dose 50 (EID_50_) challenge virus was administered intranasally to each horse by nebulization using the Flexineb^®^ (Nortev, Ireland). All procedures were performed at MSD Animal Health, the Netherlands. To ensure blinding, all personnel involved with the vaccinations were not involved in clinical observations and vice versa.

This study was performed in compliance with the Dutch Experiments on Animals Act 2014, with approval of the MSD Animal Health Ethics Review Committees and Dutch Central Authority for Scientific Procedures on Animals (license number AVD2210020172725, approved 1 November 2017).

### 2.5. Sample Collection

From all horses, blood samples were collected on days 0 (prior to vaccination), seven, 28 (week four, prior to vaccination), 49 (week seven, prior to challenge), 56 (week eight), and 63 (week nine, end of study). For separation of serum, blood samples were transported to the laboratory and stored at ≤−15 °C until testing.

From all horses, nasal swab samples were collected on days 0 and 28 (week four, prior to vaccination), day 49 (week seven, prior to challenge), and daily from day 50 to day 63 (week nine). Swabs were taken from alternating nostrils between samplings and from one nostril on each sample occasion. After sampling, swabs were placed in a transport medium (phosphate-buffered saline with fetal calf serum and antibiotics/antifungals) and stored at ≤−70 °C until testing.

### 2.6. HI Assay

Serum was tested for EIV-specific antibodies at MSD Animal Health (the Netherlands) using an HI assay as previously described [9], and HI titres were expressed as log_2_ values. A titre of >4 was considered as significant, and for graphical and statistical purposes, a titre of <4 was converted to three, and a two-fold log_2_ increase of the titre was considered indicative of seroconversion.

### 2.7. Observations of Clinical Signs

Observations of clinical signs, including rectal temperature, nasal discharge, cough, ocular discharge, dyspnea, anorexia, and depression, were assessed, recorded, and analyzed according to the scoring system in Table 1 (set-up in cooperation with experts in the equine field and used during registration of various commercial equine products). The horses were observed once daily from day 49 (week seven, prior to challenge) to 63 (week nine), and observations were performed by trained personnel. Rectal temperature was measured daily from day 49 (prior to challenge) to 63 (week nine) using a digital thermometer.

### 2.8. Shedding of Live EIV

Nasal swabs were tested for shedding of live EIV after challenge at MSD Animal Health (the Netherlands) by virus titration assay in embryonated SPF hens’ eggs, as previously described [10].

All swab samples were first screened for presence of live virus, after which only in the virus-positive samples was the amount of live virus quantified.

### 2.9. Data Analysis and Statistics

The equine influenza vaccine (inactivated) 0249 monograph [11] was used to evaluate vaccine efficacy. This monograph described that the evaluation of efficacy is based on control horses showing characteristic clinical signs, whereas vaccinated horses should only show no to mild clinical signs. The average number of days on which the virus is excreted and the virus titres should both be significantly lower in vaccinated compared to control ponies.

The statistical approach was used similarly as applied in our recent publication on the same vaccine [12]. In short, descriptive statistics were conducted to indicate the trend of vaccine efficacy, followed by inferential comparisons to show *p*-values. Rectal temperature data after challenge were evaluated using a linear mixed model analysis of variance (ANOVA) for repeated measures, including temperature measured at day 0 of challenge (baseline) as the covariate. Additionally, ANOVA was used to evaluate the difference in peak rectal temperature from baseline. Clinical and total clinical score data (the overall of the clinical score plus the rectal temperature score) over time after challenge were analyzed using generalized estimating equations (GEEs) using a cumulative logit model. Because all viral shedding values (both pos/neg scoring and titre) were negative for vaccinated animals in this study, GEE and ANOVA are not suitable models. Therefore, Fisher’s exact test was used to compare proportions of viral shedding, and the Wilcoxon rank sum test was used to compare viral shedding titres between the vaccinated and control group in the overall challenge period until the last moment of shedding in the control group was detected. This is also the reason why we did not statistically analyze the average number of days the virus was excreted. For all inferential statistical analyses, the level of significance α was set at 0.05 and tests were two-sided. Statistical software package SAS V9.4 (SAS Institute Inc., Cary, NC, USA) was used.

## 3. Results

### 3.1. HI Antibody Response

**Figure 1:**
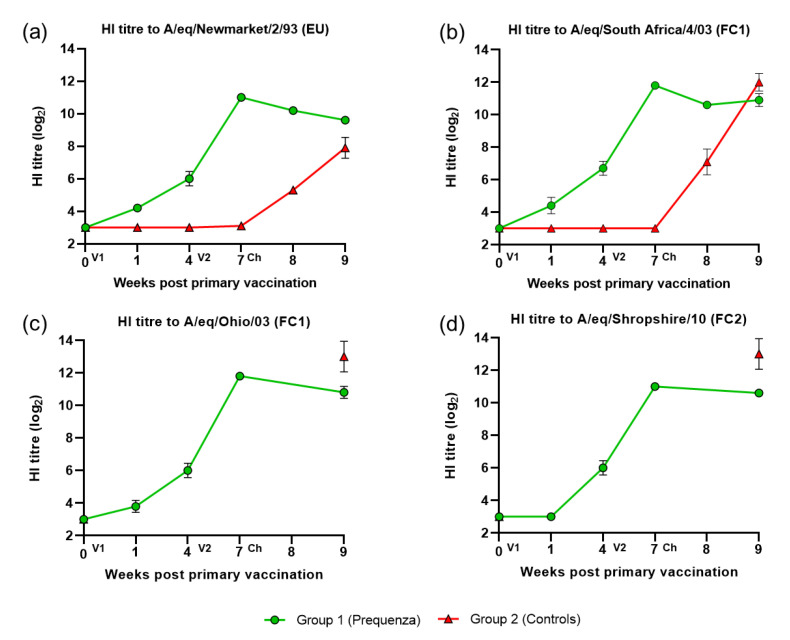
Mean hemaglutination inhibition (HI) antibody titre response in group 1 and 2 against H3N8 EIV (**a**) A/equine/Newmarket/2/93, (**b**) A/equine/South Africa/4/03 (FC1), (**c**) A/equine/Ohio/03 (FC1), and (**d**) A/equine/Shropshire/10 (FC2). Week 0, V1; Week 4, V2; Week 7, virus challenge (VC). Green square = group 1, Equilis Prequenza vaccinated group; red triangle = group 2, unvaccinated control group. Error bars represent SEM. A titre of <4 was converted to three for graphical presentation.

All horses were seronegative/low seropositive on day 0 prior to V1. After V1, HI titres to both EIV FC1 and FC2 and EU were observed in horses in the vaccinated group (Figure 1). After V2, HI titres in the vaccinated group increased up to week seven (day 49, day of challenge). After challenge, HI titres in the vaccinated group remained similar or declined only marginally (<1.5 HI titres), while in the control group, HI titres increased markedly.

### 3.2. Clinical Signs of Disease after Challenge with H3N8 A/equine/Venlo/19 (FC1 Sub-lineage)

After challenge, clinical signs of EI disease (coughing, sneezing, dyspnea, and nasal and ocular discharge) and rectal temperature were scored (Table 1) and combined to provide a clinical score and a total clinical score (clinical score plus rectal temperature score).

**Figure 2:**
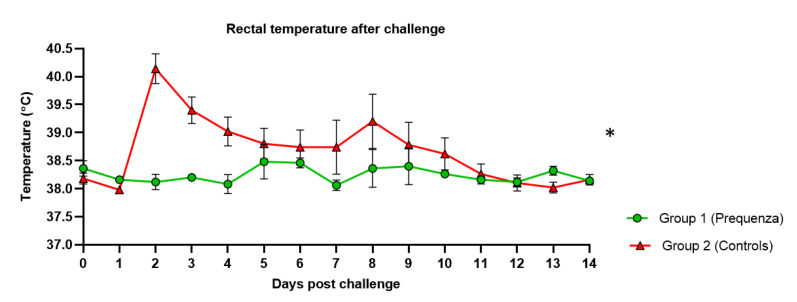
Rectal temperature in groups 1 and 2 after challenge with H3N8 A/equine/Venlo/19 (FC1 sub-lineage). Green square = group 1, Equilis Prequenza vaccinated group; red triangle = group 2, unvaccinated control group. Error bars represent SEM. Inferential statistics: significant difference (*p* < 0.05) between groups 1 and 2 (*) (mean effect between 1 and 14 dpc).

In the control group, rectal temperature post-challenge increased, peaking at two and eight days post-challenge (dpc), with an average temperature of 40.1 °C and 39.2 °C, respectively (Figure 2). In the vaccinated group, only minor elevations were observed, not exceeding an average temperature of 38.5 °C. Post-challenge rectal temperature was significantly lower (Table 2) in the vaccinated group compared to the control group (*p* = 0.0156). This was also seen in the change in rectal temperature from baseline, where the peak change in rectal temperature from baseline was significantly lower (Table 2) in the vaccinated group compared to the control group (*p* = 0.0478).

**Figure 3:**
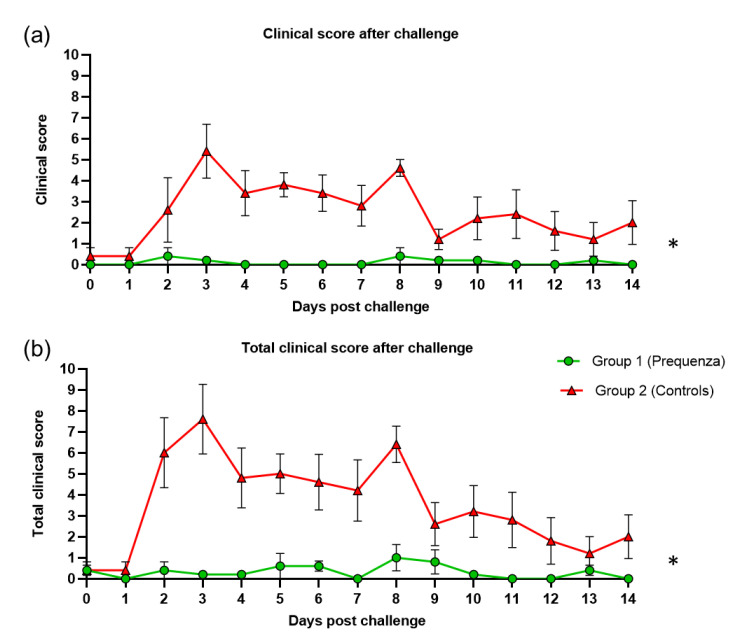
(**a**) Clinical score (clinical score excluding rectal temperature) and (**b**) total clinical score (clinical signs and rectal temperature) in groups 1 and 2 after challenge with H3N8 A/equine/Venlo/19 (FC1 sub-lineage). Green square = group 1, Equilis Prequenza vaccinated group; red triangle = group 2, unvaccinated control group. Error bars represent SEM. Inferential statistics: significant difference (*p* < 0.05) between groups 1 and 2 (*) (mean effect between 1 and 14 dpc).

Clinical signs were observed in the control group throughout the entire monitoring period after challenge, peaking at three and eight dpc, with an average clinical score of 5.4 and 4.6, respectively (Figure 3). By 14 dpc, the control group still had an average clinical score of 2.0. In the vaccinated group, no peaks were observed, only a small elevation at two and eight dpc, with a highest average clinical score of 0.4. The clinical score post-challenge was significantly lower (Table 2) in the vaccinated group compared to the control group (*p* < 0.0001) (mean effect between 1 and 14 dpc).

The total clinical score (clinical signs and temperature) in the control group peaked at three and eight dpc, with average total clinical scores of 7.6 and 6.4, respectively (Figure 3). By 14 dpc, the control group still had an average total clinical score of 2.0. In the vaccinated group, the total clinical score peaked at eight dpc, and the average total clinical score was 1.0. The total clinical score post-challenge was significantly lower (Table 2) in the vaccinated group compared to the control group (*p* < 0.0001) (mean effect between 1 and 14 dpc).

### 3.3. Virus Shedding after Challenge with H3N8 A/equine/Venlo/19 (FC1 Sub-Lineage)

**Figure 4:**
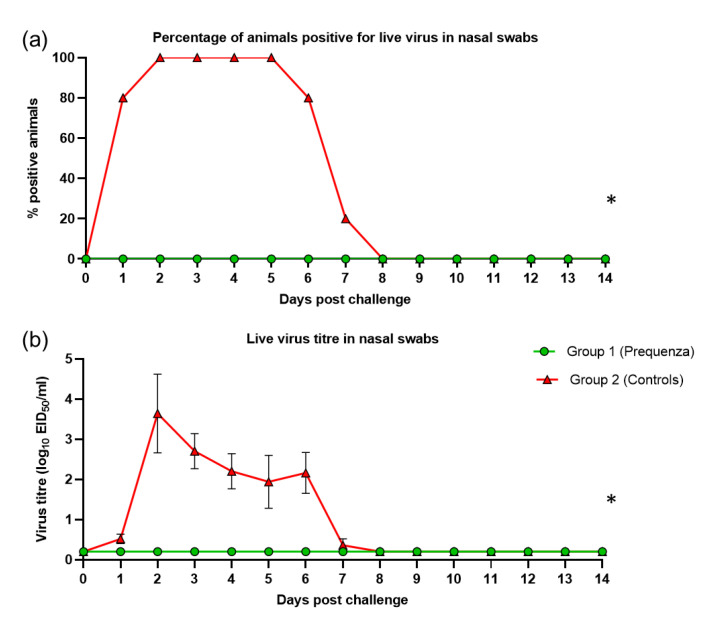
(**a**) Percentage of horses positive for live virus in nasal swabs and (**b**) Live virus titre in nasal swabs in groups 1 and 2 after challenge with H3N8 A/equine/Venlo/19 (FC1 sub-lineage). Green square = group 1, Equilis Prequenza vaccinated group; red triangle = group 2, unvaccinated control group. Error bars represent SEM. Inferential statistics: significant difference (*p* < 0.05) between groups 1 and 2 (*) (overall period one to seven dpc).

Shedding of live virus after challenge was evaluated by the percentage of horses positive for live virus and mean live virus titres. Shedding of live virus was not detected in any of the vaccinates (0%), but was present in all five control horses (100%) at 2–5 dpc (Figure 4). Virus titres in nasal swabs increased in the control group, peaking at two dpc with an average titre of 3.6 (Figure 4). All control horses had ceased shedding live virus by eight dpc. Therefore, interferential statistics were only performed between one and seven dpc. Each of the viral shedding responses (the odds of a positive sample and the virus titre) were significantly lower (Table 2) in the vaccinated group compared to the control group (*p* < 0.0001).

## 4. Discussion

In this study, the efficacy of a commercially available ISCOMatrix-adjuvanted, whole inactivated EIV vaccine (Equilis Prequenza) in protecting against challenge with an isolate of the recent FC1 outbreak strain was evaluated. Serological responses, clinical signs, and virus excretion were evaluated after experimental challenge infection three weeks after the primary vaccination course (V1 and V2) in horses and compared to unvaccinated control horses.

Following experimental infection, rectal temperature and clinical signs were markedly lower in the vaccinated group compared to unvaccinated controls. In addition, virus excretion was absent in the vaccinated group, while from 1–7 dpc the virus was excreted by the unvaccinated controls, indicating that sterile immunity was induced in the vaccinates. This was an interesting finding, since field experience with this new EIV FC1 strain as reported from France was that several outbreaks had involved correctly vaccinated animals, the vast majority vaccinated with a vaccine fully in line with OIE recommendations [4,7]. One of the main differences between the predominant vaccine used in France and Equilis Prequenza is that the former only expresses HA, while the latter contains all viral proteins, as it is a whole inactivated virus vaccine. Results in mice showed that a whole inactivated influenza virus vaccine provided a stronger antiviral and more Th1-skewed immune response of dendritic cells in vitro and superior immunogenicity in vivo, providing better protection than subunit vaccines containing/expressing only some of the viral proteins [13,14]. Furthermore, cross-protection against heterologous virus strains appears to be primarily mediated by CTLs rather than by antibodies in mice [15]. CTLs are mainly directed at internal viral proteins like nucleoprotein (NP) [16,17,18], which is present in a whole inactivated vaccine, but absent in a subunit vaccine containing/expressing only HA and/or NA. The ability of a whole inactivated virus vaccine and the inability of a subunit vaccine to induce a specific CTL response was correlated to cross-protection in mice [15]. This may be a possible explanation for some of the differences in the immunity observed in this study and the immunity observed in the field in horses vaccinated with subunit vaccines during the FC1 outbreaks in 2018/19.

## 5. Conclusions

In conclusion, this study shows that Equilis Prequenza provides a high level of protection following challenge with the recent EIV FC1 outbreak strain, resulting in a clear reduction of clinical signs and prevention of excretion of the challenge virus. Furthermore, this suggests that, aside from antigenic differences between the vaccine and field strain, the nature of the antigen and/or adjuvant may likely play an important role in vaccine efficacy against new field strains, as previously described [12]. However, further research is needed in these aspects to investigate the role of antigenic differences between vaccine and field strain and other attributes of EIV vaccines, such as vaccine type or adjuvant, in performance of the EIV vaccine in the field.

## Figures and Tables

**Table 1:** Scoring system for observation of clinical signs.

Clinical Observation		Score
General Health	No abnormality found	0
	Diarrhea	1
	Malaise/depression/normal eating	1
	Malaise/depression/reduced appetite	2
	Dehydration	2
	Anorexia	4
	Colic	5
	Down/unable to stand	30
	Dead	100
Respiratory	Hyperpnoea	2
	Dyspnea	4
	Cough/2–5× in 10 min	1
	Cough/6–20× in 10 min	2
	Cough/>20× in 10 min	3
Ocular	Lachrymation	1
	Mild mucopurulent discharge	2
	Marked mucopurulent discharge	4
	Mild conjunctivitis	2
	Marked conjunctivitis	4
Nasal	Nasal serous discharge	1
	Mild nasal mucopurulent	2
	Marked nasal mucopurulent	4
	Sneeze/2–5× in 10 min	1
	Sneeze/6–20× in 10 min	2
	Sneeze/>20× in 10 min	3
Temperature (°C)	<38.5	0
	38.5–39.0	1
	39.1–39.5	2
	39.6–40.0	3
	>40.0	4

**Table 2:** Inferential statistics: comparison of the rectal temperature, clinical observations, and viral shedding between groups after challenge (mean effect between 1 and 14 dpc ^a^ or 1 and 7 dpc ^b^).

	Group Comparison 1 = Control, 2 = Equilis Prequenza
Parameter	2 vs. 1
Rectal temperature over time ^a^	0.0156
Peak change rectal temperature post-challenge ^a^	0.0478
Clinical score ^a^	<0.0001
Total clinical score ^a^	<0.0001
Viral shedding nasal swabs +/− ^b^	<0.0001
Viral shedding nasal swab titre ^b^	<0.0001

^a^ effect between 1 and 14 dpc. ^b^ effect between 1 and 7 dpc. + nasal swabs positive for EIV. − nasal swabs negative for EIV.
